# Quantification of an External Motion Surrogate for Quality Assurance in Lung Cancer Radiation Therapy

**DOI:** 10.1155/2014/595430

**Published:** 2014-11-30

**Authors:** Jens Wölfelschneider, Tobias Brandt, Sebastian Lettmaier, Rainer Fietkau, Christoph Bert

**Affiliations:** ^1^University Hospital Erlangen, Radiation Oncology, Universitätsstraße 27, 91054 Erlangen, Germany; ^2^Friedrich-Alexander-University Erlangen-Nürnberg (FAU), Faculty of Medicine, Krankenhausstraße 12, 91054 Erlangen, Germany; ^3^GSI—Helmholtz Centre for Heavy Ion Research, Department of Biophysics, Planckstraße 1, 64291 Darmstadt, Germany

## Abstract

The purpose of this work was to validate the stability of the end exhale position in deep expiration breath hold (DEBH) technique for quality assurance in stereotactic lung tumor radiation therapy. Furthermore, a motion analysis was performed for 20 patients to evaluate breathing periods and baseline drifts based on an external surrogate. This trajectory was detected using stereo infrared (IR) cameras and reflective body markers. The respiratory waveform showed large interpatient differences in the end exhale position during irradiation up to 18.8 mm compared to the global minimum. This position depends significantly on the tumor volume. Also the baseline drifts, which occur mostly in posterior direction, are affected by the tumor size. Breathing periods, which depend mostly on the patient age, were in a range between 2.4 s and 7.0 s. Fifteen out of 20 patients, who showed a reproducible end exhale position with a deviation of less than 5 mm, might benefit from DEBH due to smaller planning target volumes (PTV) compared to free breathing irradiation and hence sparing of healthy tissue. Patients with larger uncertainties should be treated with more complex motion compensation techniques.

## 1. Introduction

Treatment of lung tumors is challenging in radiation therapy due to the presence of intrafractional motion. Respiration and organ motion result in tumor displacement, which has to be regarded and compensated. Uncertainties in target position are often considered by an expansion of the clinical target volume (CTV) [1]. More CTV-conformal treatments can nominally be achieved with treatment protocols such as* gating* [2],* tracking* [3], or* breathing maneuvers* [4, 5] all aiming at a reduction of the effective motion amplitude. One of such methods, which do not need any further enlargement of the CTV with large safety margins, is an active or passive breath hold technique [6, 7]. A deep expiration breath hold (DEBH) approach is applied at the University Hospital in Erlangen for stereotactic treatment of bronchial carcinoma and metastasis. The end exhale phase is recommended for breath holding treatment due to higher reproducibility and stability [8].

Setup errors can further increase target failure during irradiation [9]. The ExacTrac system (Brainlab AG, Feldkirchen, Germany) matches daily kV images to digitally reconstructed radiographs (DRR) generated from the planning computed tomography (CT) to offer a high quality of patient positioning and reduction of the setup errors [10]. Furthermore, the system is able to track infrared (IR) reflective external body markers, which can be positioned on the sternum of the patients [11]. The resulting surrogate can be used to validate the end exhale position during radiation delivery.

The scope of this study was to analyze these surrogates for quality assurance and to investigate the reproducibility of the end exhale position. In addition, several parameters of the motion pattern were quantified such as motion period and baseline drifts.

## 2. Material and Methods

### 2.1. Patient Data

Retrospective data of 20 patients between 33 and 80 years with bronchial carcinoma and metastasis were analyzed for this study. Details including specific patient data are summarized in Table 1. These patients were treated at the University Hospital in Erlangen during June 2013 and April 2014 with stereotactic body radiation therapy (SBRT) [12] in combination with DEBT using the 6 MV photon beam of a Novalis machine (Brainlab AG, Feldkirchen, Germany). Breath hold was communicated to the patients by commands of the therapist who checked by video screen monitoring if the instructions were followed by the patient before activating the beam. A hypofractionated protocol of 12 × 6 Gy was used with 3D conformal treatment plans, resulting in a total number of more than 200 measurements included in this study. Treatment planning was done based on an end exhale CT provided by a Siemens Sensation Open CT scanner (Siemens, Erlangen, Germany) using a slice thickness of 3 mm. The PTV was defined as a union between gross tumor volume (GTV) in end exhale phase and mid ventilation plus a 5 mm safety margin.

### 2.2. ExacTrac

The ExacTrac v5.5.2 system was used for patient positioning [10] before treatment to minimize setup errors. This system has two orthogonal X-ray tubes which are used to match daily stereoscopic kV images to DRRs from the planning CT scan. The system is also equipped with an optoelectronic motion tracking function by means of stereo IR cameras and IR-reflecting body markers. A single marker positioned close to the sternum of the patients in each fraction was used to measure the respiratory motion with a frequency of 20 Hz as an external surrogate.

To correlate the marker position to the beam-on times and consequently to analyze the motion surrogate for each irradiation field, a Geiger counter was used to generate a beam status signal (*GM-10*, Black Cat Systems, Westminster, USA). Superposition of the beam on/off signal and the IR-marker motion trace was done manually by matching the beam-on states to the end exhale breath hold phases.

### 2.3. Data Analysis

Data analysis focused on the respiratory waveform in anterior-posterior (A-P) direction. Minima and maxima of this signal were automatically determined for the free breathing phases based on an algorithm reported by Lu et al. [13]. These points were automatically corrected for two consecutive minima or maxima as well as for periods smaller than 1/20 of the mean period. The position of the body marker is relative to the ExacTrac system's reference coordinate and therefore depends on the patient positioning. As a consequence, the absolute values were normalized by subtracting the global minimum of each fraction from each measured data point.

As knowledge of the patient's position during irradiation is necessary for an investigation of reproducibility and also for estimating dosimetric effects, a mean position of the IR marker* Δ* was computed for each breath hold phase. Breathing periods *T* are also an important motion parameter as they might influence the delivered dose to intrafractionally moving targets, so they were calculated for each breathing cycle considering only measured data in beam-off phases. In addition, baseline drifts *D* of the exhale position are a major parameter for several correlation models for internal/external motion, for example, reported in Fassi et al. 2014 [14] and estimated in this study by linear regression of the automatically detected minima. The drift was then calculated by the difference of the resulting regression function at the end and beginning of each treatment session. The analysis was done for each fraction of each patient separately to validate the stability of the breathing pattern as well as for the total treatment of all patients.

As the results might be affected by the patients' condition,* Δ*, *T*and *D* were also investigated in dependency of the patient age at treatment time as well as for the tumor volume. Boxplots were created and corrected for outliers, including median, minima, and maxima plus lower and upper quartile. A linear model was calculated to obtain the analysis for statistical significance and the coefficient of variation (CV) was determined using the quotient of standard deviation of each parameter to its mean value.

## 3. Results

**Figure 1:**
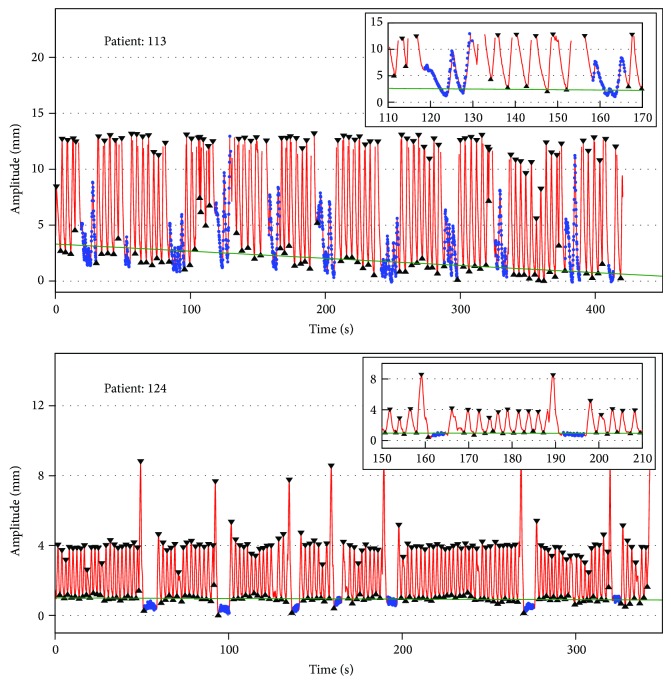
Two representative examples for the respiratory waveform detected by the ExacTrac System. The IR marker position is illustrated in red. Automatically detected minima and maxima are shown with black triangles. The blue points represent the position during the beam-on state. The baseline, calculated by linear regression of the minima, is shown by a green line. A 60 s enlargement including two breathing cycles is shown for both patients on the upper right side.

Two representative examples of the respiratory waveform for one treatment fraction are shown in Figure 1. The lower image shows a regular breathing pattern over the whole session of typically 360–600 s. The breath hold phases, which were detected using the Geiger counter signal, are always at the end exhale position. A deep inspiration is visible before each irradiation field for all analyzed patients, meaning that patients are trying to fill their lungs before breath holding. A baseline drift is also not discernible for this patient. The breathing pattern for the upper case looks completely different. The exhale position shows a high variation within this fraction and also the position during irradiation is spread over the whole breathing cycle (blue dots), indicating that the breathing maneuvers are not sufficiently fulfilled by this particular patient. Consequently the breath hold phases are not stable and spread from 0.5 to 4.9 mm compared to the global minimum. A drift of the exhale position of −2.1 mm from the beginning to the end of treatment is also visible for this patient. Missing lines, which are visible in the enlargement of the upper image, represent missing signals from the ExacTrac system.

**Figure 2:**
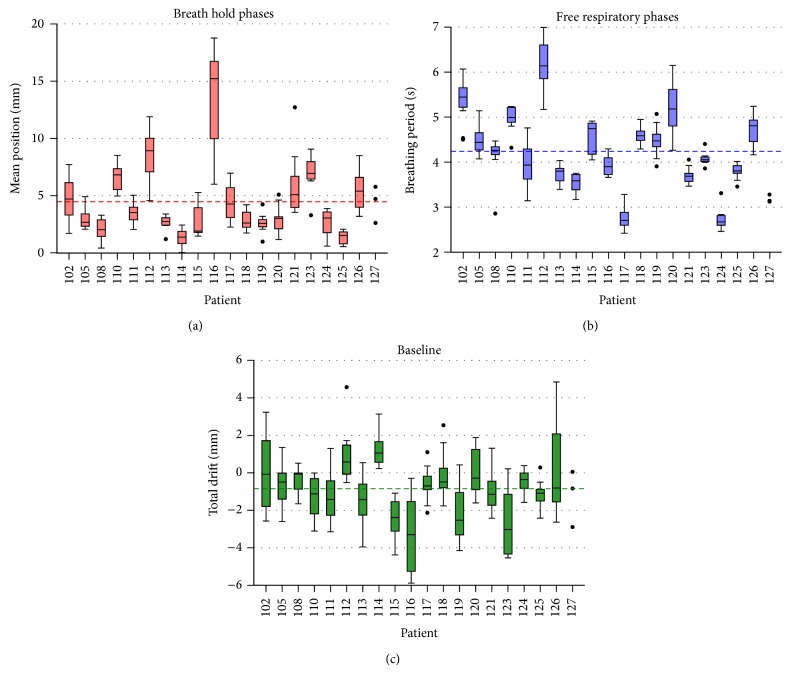
Boxplots for the mean position of the breath hold phases* Δ* (red), breathing periods *T* (blue), and baseline drifts* D* (green) for all patients and all fractions. The mean value is marked by a colored dashed line. Outliers are illustrated with black dots.

The results for all patients are illustrated in Figure 2. The breathing periods show the highest interpatient variations ranging from 2.4 to 7.0 s with a mean of 4.24 s and a mean coefficient of variance of ${\overset{-}{\text{CV}}}_{T}=0.22$. However, intrapatient variations in between individual treatments are quite smaller (${\overset{-}{\text{CV}}}_{T,\;\text{Patient}}=0.07$). The coefficient of variance for the mean amplitude and the baseline drift are ${\overset{-}{\text{CV}}}_{\Delta }=0.75$ and ${\overset{-}{\text{CV}}}_{D}=2.72$, respectively. 73% of all measured baseline drifts are negative with a mean of $\overset{-}{D}=-0.85\;\text{mm}$. The mean position of the breath holding phases Δ is spread from 0.1 to 18.8 mm relative to the global minimum with an overall mean of $\overset{-}{\Delta }=\text{4.48}\;\text{mm}$.

**Figure 3:**
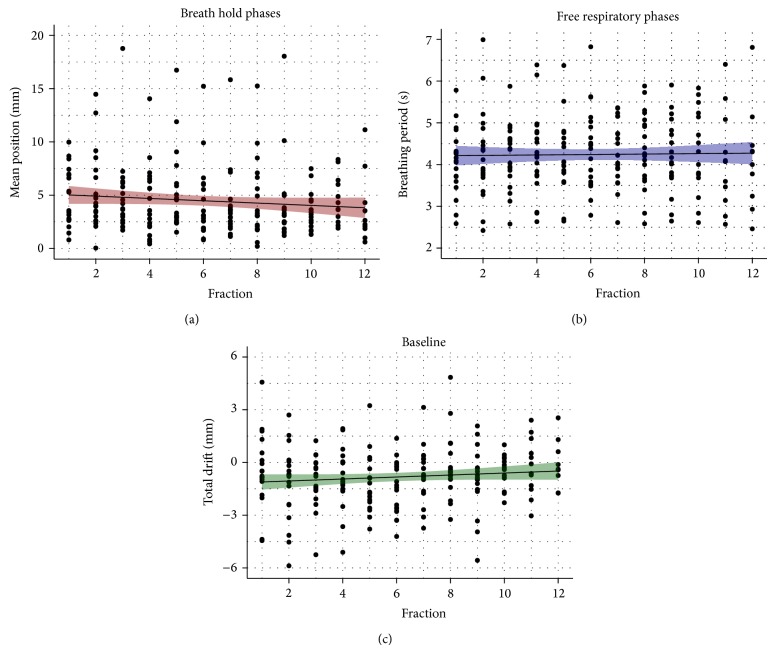
Results of the mean position of the breath hold phases* Δ* (red), breathing periods *T* (blue), and baseline drifts* D* (green) over the number of fractions. Each point represents a measurement for one patient in one fraction. The colored area represents the 95% confidence interval of the linear regression (black line).

The analysis for each individual fraction is shown in Figure 3. The position of the breath hold phase seems to decrease slightly but not significantly for an increasing number of fractions (*p* = 0.125). Also breathing periods are constant in a first instance over the whole treatment course and consequently do not depend on the number of fractions (*p* = 0.783). For the baseline drifts a small but highly significant positive tendency is visible (*p* = 0.007), meaning that these drifts might be reduced after several fractions.

**Figure 4:**
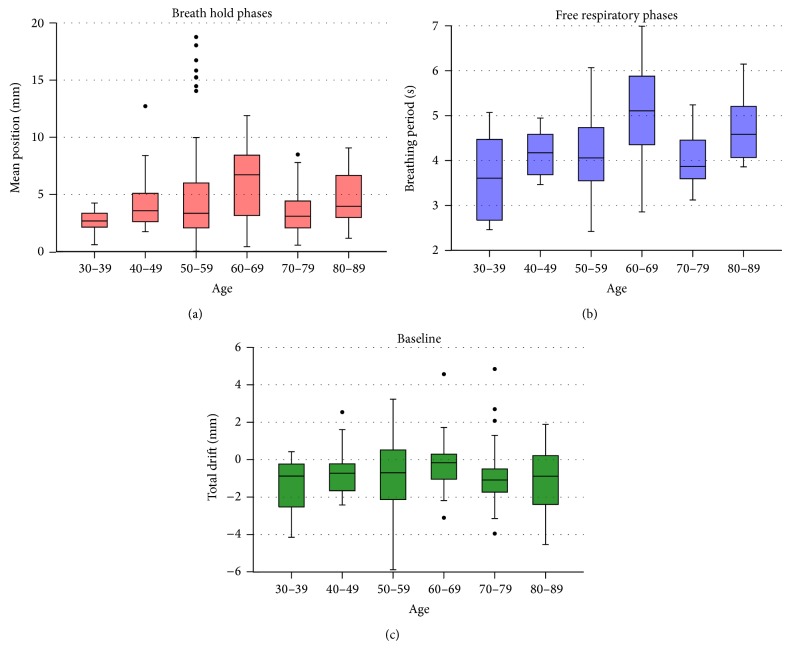
Boxplots for the mean position of the breath hold phases* Δ* (red), breathing periods *T* (blue), and baseline drifts* D* (green) in dependency of the patient age at treatment. Outliers are illustrated with black dots.

As the analyzed parameters show a high interpatient variation, the further analysis was further focused on the patient specific properties.  Figure 4 shows the results in dependency of the patient age. Even though a rising tendency is visible for the mean breath hold positions* Δ*, indicating that uncertainties increase with an increasing age, the trend is not statistically significant (*p* = 0.122). Also baseline drifts are not effected from the patient age and show a nearly constant mean value (*p* = 0.682). Only breathing periods increase statistically significant (*p* < 0.001) for older patients, meaning that older patients breath calmer or deeper than younger ones.

**Figure 5:**
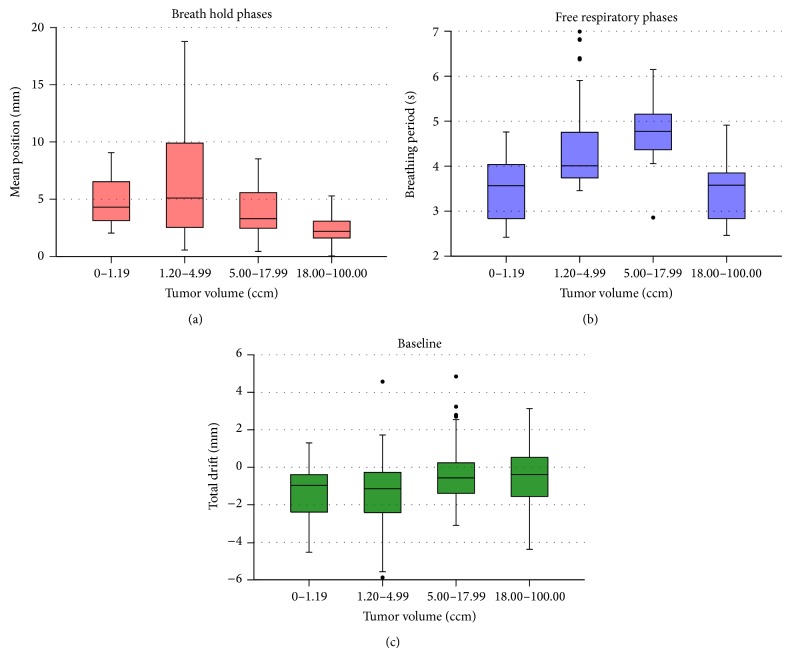
Boxplots for the mean position of the breath hold phases* Δ* (red), breathing periods *T* (blue), and baseline drifts *D* (green) in dependency of the tumor volume. Outliers are illustrated with black dots.

The results for the dependency of the tumor volume are shown in Figure 5 for all analyzed parameters. Here, the breath hold position Δ as well as the baseline drift *D* depends statistically significantly on the tumor volume (*p* < 0.001). The scattering for breathing periods is much larger resulting only in a small but not visible interference of the tumor size (*p* = 0.028).

## 4. Discussion

The respiratory waveform represented by a chest wall surrogate was analyzed for 20 patients with lung tumors. Several parameters, including breathing periods and baseline drifts, were examined for each fraction and for the total treatment. Furthermore, the stability and reproducibility of the end exhale position were investigated using an external surrogate detected by the ExacTrac system.

This system is able to locate the body markers with submillimeter accuracy in dependency of the isocenter but is also susceptible for missing signals (see Figure 1) due to IR markers which are outside of the field of view especially in case of couch rotations. Since the main purpose of the ExacTrac version used in this study is patient positioning and correction, the tolerances for a lateral shift have to be less than 10 mm. In case of tracking markers for motion analysis which is, for example, incorporated in the ExacTrac version used for the* vero *SBRT system [15], the 10 mm restriction seems to be counterproductive due to the fact that body markers should also be tracked after couch rotations and consequently after changes of the initial marker geometry of more than 10 mm. A third infrared camera may at least solve the problem of hidden markers after couch rotation.

Treatments using a deep expiration breath hold technique lead to smaller target volumes and sparing of healthy tissue in contrast to a free breathing irradiation without any further motion compensation methods. The respiratory waveforms have shown that breath holding for up to 15 s during irradiation was not a real problem for all analyzed patients and could be applied easily. The reproducibility of this irradiation phase still varied substantially. Nevertheless, 71% of the patients revealed a reproducible breath hold position with a deviation of smaller than 5 mm. For patients with a larger scattering of the exhale position, the breath hold technique might not be successful, so additional methods are necessary. This subgroup may benefit from other motion compensation techniques such as tracking [16] or gating [2], which could be applied under free breathing.

Furthermore, younger patients might be more appropriate for breath holding treatments than older ones as the exhale position seems to be more reproducible, but one respiratory cycle is faster (see Figure 4). Breathing periods are a major parameter of motion analysis due to a direct effect on the tumor position, so they have to be taken into account especially in case of free breathing treatment concepts. All investigated periods of 2.4 to 7.0 s are comparable to other studies, for example, from Seppenwoolde et al. [17], and are stable during the whole treatment of each patient (see Figure 3). To further increase the stability of a specific breathing phase, patient training using audio or visual feedback [18, 19] could be one aspect to improve multiple breath holds in several fractions.

The study has also shown that baseline drifts are mostly negative, indicating a sag of the patient towards the couch, which might be due to a more relaxed situation of the patient at the end of treatment. These posterior drifts are usually smaller than 1 mm but can be up to 6 mm and therefore have to be considered for treatment planning and target volume definition.

The results have also provided that the tumor volume might have a direct effect on the baseline drift and the exhale position (see Figure 5). One possible reason might be that the peak-to-peak breathing amplitude decreases for patients with larger tumors, so consequently the end exhale position also decreases. To confirm this statement, further investigations have to be done including a correlation between a normal breathing behavior and the tumor volume of each specific patient.

The whole analysis was based on the infrared cameras from the ExacTrac system, which seems to work very precisely and suitably for clinical practice. Nevertheless, the system measures an external surrogate only, which has to be correlated to the internal tumor motion to estimate dosimetric effects. The analysis for the internal motion can be done based on multiple 4DCTs that were obtained as part of the clinical protocol and are currently under investigation. However, the 4DCT rely also on an external signal to arrange each motion phase. In addition, the surrogate was only used to define the breath hold position in relation to the global minima. As a consequence, breathing periods and baseline drifts can be assumed by the external surrogate.

## 5. Conclusion

This study reports a motion analysis of an external surrogate using the ExacTrac system to validate the stability of breath hold technique. Respiratory waveforms differ for the investigated patients. Deviations of up to 19 mm can be expected but only 29% of the patients show variations of the end exhale position more than 5 mm. Most of the patients may benefit from the simplicity of breath holding, but some patients with an irregular breathing pattern should be treated with more complex motion compensation techniques. The baseline drift of up to ±6 mm should be considered for treatment planning.

## Figures and Tables

**Table 1:** Patient data, including age at treatment time, sex, entity plus stage of primary tumor and the treated tumor volume in ccm.

Patient	Age	Sex	Entity	UICC	Stage	Tumor volume [ccm]
Anonymous 102	58	f	Sarcoma	IV	cT2 cN0 cM1	12.430
Anonymous 105	56	m	CUP	IV	*not available *	8.467
Anonymous 108	64	m	NSCLC	IIIa	cT3 cN2 M0	5.963
Anonymous 110	63	m	Prostate-Ca	IV	pT3b pN1 cM1	9.117
Anonymous 111	75	m	Sigma-Ca	IV	T2 pM1	0.819
Anonymous 112	68	m	SCLC	IV	cT2 cN2 pM1	3.829
Anonymous 113	71	m	NSCLC	IV	cT3 cN3 cM1b	18.083
Anonymous 114	50	m	Sarcoma	IV	ypT2b pM1	55.317
Anonymous 115	50	f	NSCLC	IV	cT2 cN3 cM1	31.357
Anonymous 116	56	m	HNSCC	IVc	cT1 cN3 cM1	1.213
Anonymous 117	51	m	Rectum-Ca	IV	cT3 cN+ cM1	1.137
Anonymous 118	44	m	Melanoma	IV	pT4b M1c	17.514
Anonymous 119	33	m	NSCLC	IIIb	cT1 cN3 cM0	3.431
Anonymous 120	80	m	NSCLC	IIIb	cT4 cN2 cM0	7.205
Anonymous 121	45	m	NSCLC	IV	cT2 cN2 cM1	4.167
Anonymous 123	80	m	NSCLC	IV	cT3 cN1 cM1	0.503
Anonymous 124	38	f	Rectum-Ca	IV	cT4 cN0 cM1	21.483
Anonymous 125	78	m	NSCLC	IV	cT4 cN2 cM1	1.333
Anonymous 126	75	m	Rectum-Ca	IV	cT3 cN+ cM1	5.316
Anonymous 127	72	m	HNSCC	IVc	pT4 pN2 pM1	0.449
